# Surgical Management of Thoracolumbar Scoliosis Secondary to Hip Joint Ankylosis and Severe Pelvic Obliquity

**DOI:** 10.7759/cureus.19744

**Published:** 2021-11-19

**Authors:** Chizuo Iwai, Kazunari Fushimi, Satoshi Nozawa, Koki Kato, Takaki Miyagawa, Iori Takigami, Haruhiko Akiyama

**Affiliations:** 1 Department of Orthopaedic Surgery, Gifu University Graduate School of Medicine, Gifu, JPN; 2 Department of Orthopedic Surgery, Gifu Prefectural General Medical Center, Gifu, JPN

**Keywords:** hip spine syndrome, leg length discrepancy, adult spinal deformity, pelvic obliquity, articular tuberculosis, ankylosed hip

## Abstract

We report a rare case of a rigid spinal deformity with severe pelvic obliquity (PO) resulting from hip ankylosis caused by childhood tuberculosis (TB). A 66-year-old woman presented with left knee pain, chronic low back pain, and fatigability during walking. She presented with leg length discrepancy (LLD) due to an ankylosed right hip joint, severe PO, and secondary lumbar scoliosis. Total hip arthroplasty (THA) and adductor tendonectomy were performed prior to spine surgery, and posterior spinal correction and fusion were performed from T10 to the pelvis. Prior to spinal correction surgery, we predicted that it would be impossible to make the pelvis perfectly horizontal. Therefore, we positioned a prosthetic acetabular cup at a small inclination angle at the upper limit of anteversion; spinal correction and fusion were then performed. Her symptoms including fatigability during walking resolved and the sagittal spinal balance on standing improved dramatically. The preoperative and postoperative values of the thoracolumbar Cobb angle was 40° and 25°, lumbosacral Cobb angle was 60° and 14°, C7 plumb line shift was 24 and 0 mm, pelvic tilt was 15° and 19°, lumbar lordosis (LL) was 23° and 60°, pelvic incidence minus lumbar lordosis (PI-LL) was 38° and 1°, the sagittal vertical axis was 80 and 0 mm, and PO was 28° and 15°, respectively. We present a case of rigid spinal deformity accompanied by hip joint ankylosis and PO. Performing THA prior to spinal correction surgery is an alternative and feasible option for the treatment of this challenging pathology.

## Introduction

The spinal kyphotic deformity is a well-known sequela of spinal tuberculosis (TB). Although spinal involvement occurs in less than 1% of patients with TB infection [1], TB arthritis of the hip constitutes approximately 15% of all cases of osteoarticular TB and is the second most frequent site of bone involvement following the spine [2]. Recent studies have reported on the safety of total hip arthroplasty (THA) with extensive debridement and appropriate anti-tubercular treatment in cases of old, non-active, or even active TB hip [3]. Advancements in surgery for TB have decreased the patient population suffering from coronal imbalance secondary to hip ankyloses in developed countries. This rarely provides the opportunity to treat secondary coronal and sagittal imbalances induced by neglected TB hip, and previous reports on this issue are lacking.

Surgical treatment of a patient with coexisting hip joint degeneration, pelvic obliquity (PO), and structural rigid spinal deformity is complicated and challenging [4]. Surgeons are often faced with a dilemma on whether to prioritize surgical treatment of the hip or spine in patients with such pathologies. Many surgeons have proposed spinal correction surgery first prior to THA [5-7]. If THA is performed first, the following adult spinal deformity (ASD) surgery significantly changes pelvic parameters including the pelvic tilt (PT); this may influence acetabular cup orientation and induce dislocation of the hip prosthesis [8]. Other authors argue that hip surgery should be performed prior to spine surgery in cases of flexion contractures of the hip [9]. However, this remains controversial. Here, we present the case of a patient who underwent surgical treatment for a rigid spinal deformity accompanied by hip joint ankylosis and PO.

## Case presentation

**Figure 1:**
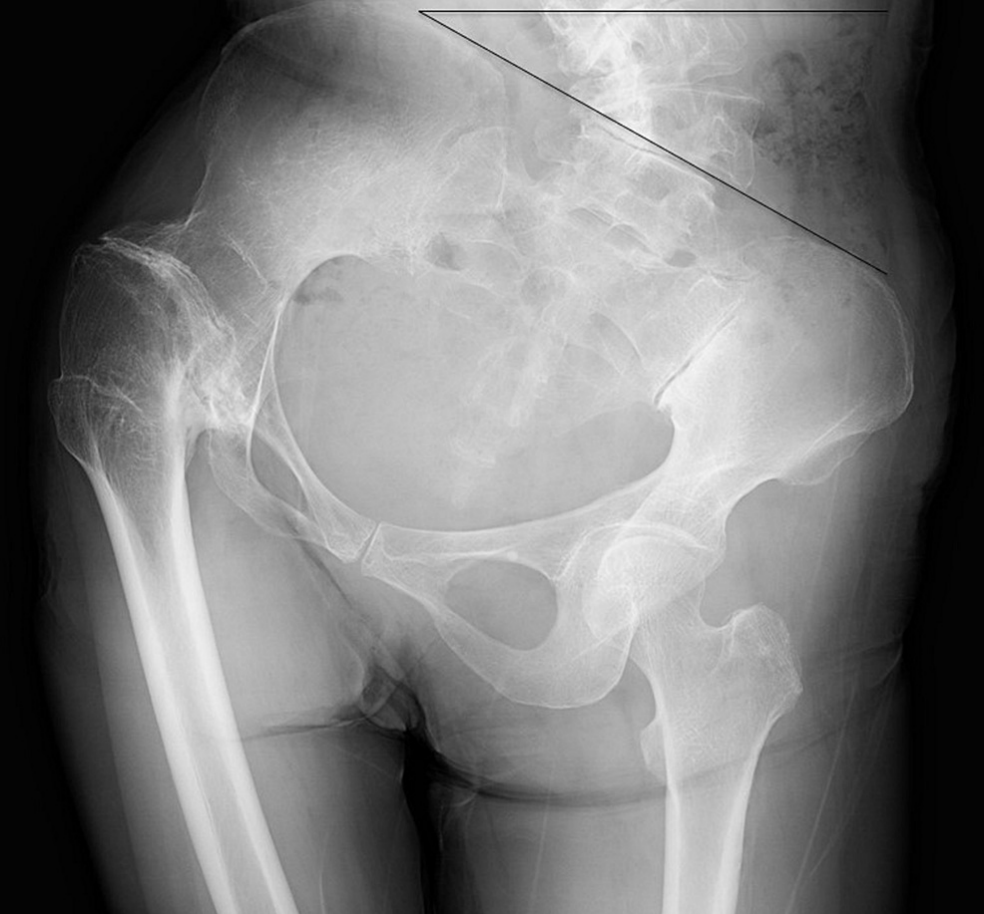
Preoperative antero-posterior radiographs of the hip joints. Preoperative antero-posterior radiographs show narrowing of the hip joint on the right side, caused by TB, with significant pelvic obliquity and adduction contracture of the hip.

A 66-year-old woman with TB hip fused in childhood complained of severe contralateral knee pain and intense fatigability during walking accompanied by leg length discrepancy (LLD), severe PO, and secondary scoliosis for the past 10 years. She underwent right hip arthrodesis for TB arthritis with antibiotics, and the affected hip joint was completely inactivated. Owing to a 20° flexed-adducted and ankylosed hip on the right side, high ipsilateral PO occurred with secondary lumbosacral scoliosis on the left convex side (Figure 1).

**Figure 2:**
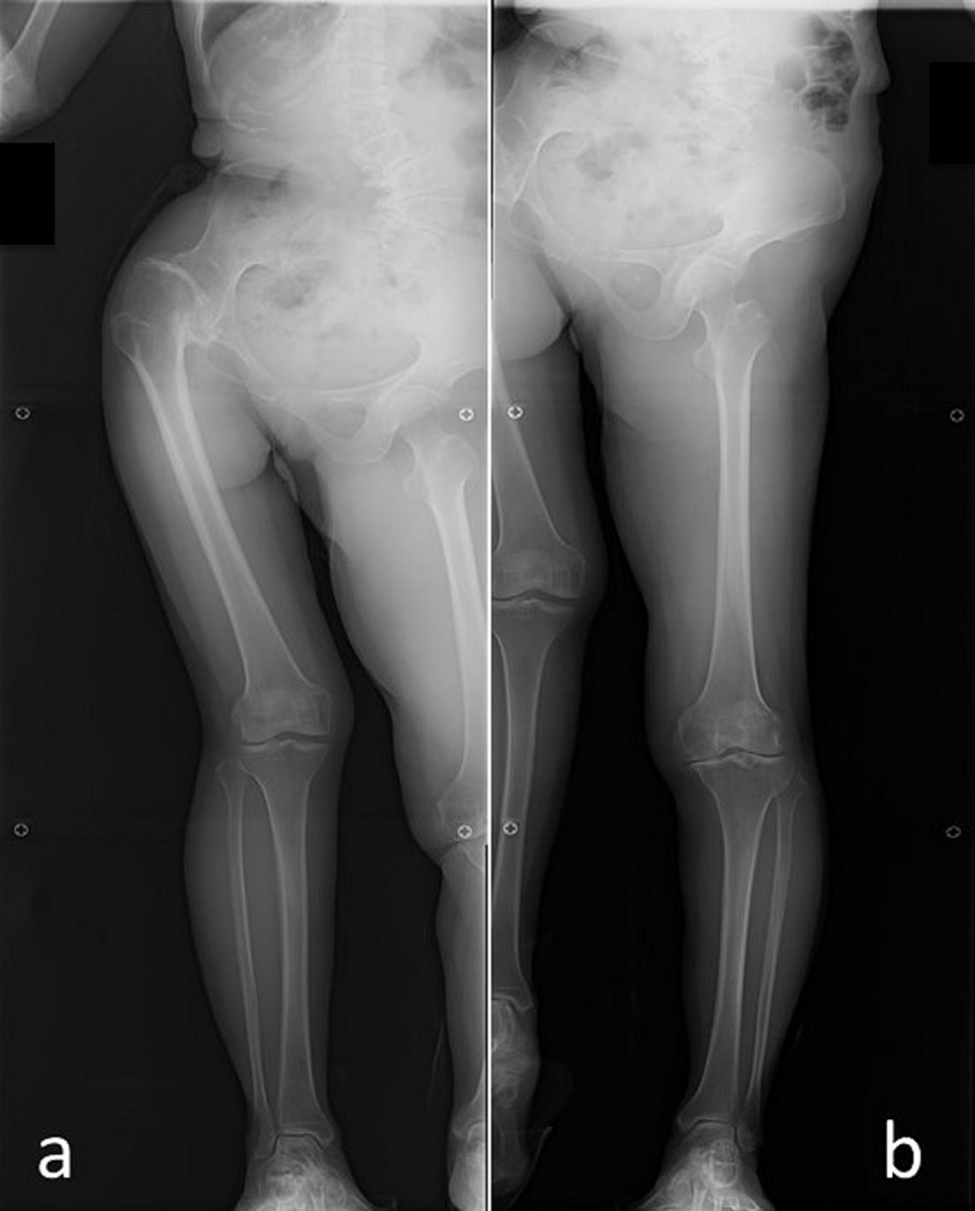
Antero-posterior radiographs of the lower extremities in standing postures. Natural standing posture represents excessive knee flexion of the longer (left) leg to adjust for the LLD (a). On the complete extension of the left knee, she was forced to stand on the toes of her affected right foot, causing plantar flexion of the foot of the shorter limb (b).

**Figure 3:**
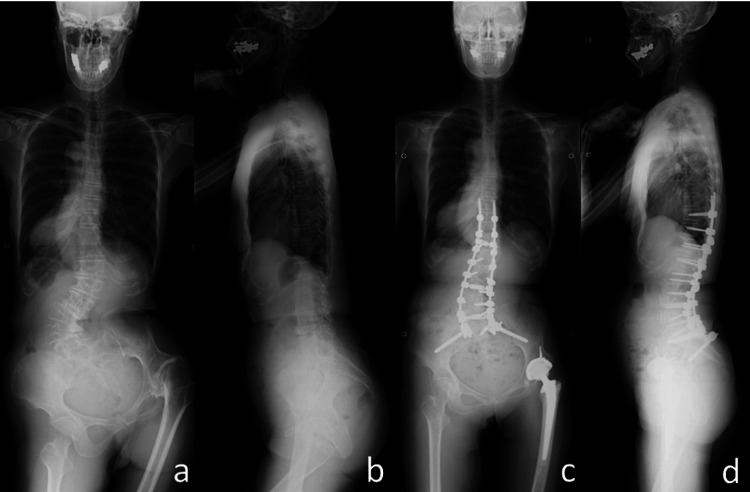
Preoperative and postoperative whole-spine posteroanterior radiographs. Patient standing supported by the entire soles of bilateral feet. Preoperative whole-spine posteroanterior radiographs showing rigid lumbosacral and thoracolumbar scoliosis and severe PO accompanied by ankylosis of the TB-affected hip on the right side (a). Preoperative lateral radiograph showing loss of lumbar lordosis and hip flexion contracture (b). The final radiograph shows almost normal alignment on the sagittal and coronal planes, significant improvement of PO, and adduction hip contracture (c and d).

The spina malleolar distance on the affected side was 2 cm shorter than that on the contralateral side. The natural standing posture represented excessive knee flexion of the longer leg to adjust for the LLD (Figure 2a-2b). If the left knee was completely extended, she was forced to stand on the toes of the affected right foot, indicating plantar flexion of the foot of the shorter limb (Figure 2b). Whole lower limb antero-posterior radiographs standing on one side showed osteoarthritis of the left knee and a change in PO (Figure 2b). Moreover, preoperative Cobb angles of the right thoracolumbar and left lumbosacral scoliosis were 40° and 60°, respectively (Figure 3a-3b). Spinal flexibility on bending or traction radiographs was limited to 30° and 53°, respectively, indicating an almost spontaneously fused spine (data not shown). The preoperative sharp angle was 34° on the affected side and 33° on the contralateral side, respectively.

**Figure 4:**
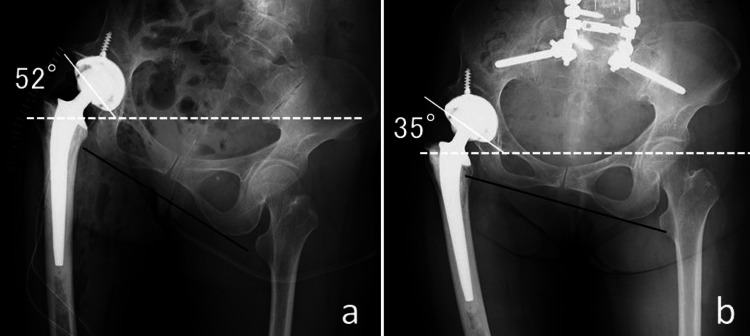
Radiographs of the hip joints before and after spinal correction surgery. After spinal correction surgery, the inclination angle for the horizontal line has changed from 52° to 35°, whereas the angle for the line connecting both teardrops is stable.

We decided to manage the ankylosed hip prior to the rigid spine deformities, without directly treating the osteoarthritis of the knee. We speculated that perfect correction of her spinal deformity was impossible. It may have been difficult to make the pelvis horizontal position; we, therefore, aimed to place an acetabular cup at 25° anteversion and 30° inclination, which was considerably less than the ideal 45° normal cup placement appropriate for a PO of 28° (Figure 4a). THA was performed through the Hardinge approach followed by adductor tendonectomy. The acetabular cup size was 50 mm and the ball size 32 mm, so call large diameter ball. After implantation, the intraoperative range of motion was 35° of flexion, 10° of extension, and 0° of abduction. Moreover, soft tissue releases around the hip joint including adductor tendonectomy improved the range of abduction from 0° to 10°.

Posterior correction and fusion from T10 to the pelvis were performed with autologous iliac bone and allografts at one week after THA, with the anterior release (Figure 3c-3d). The surgeries were successful without any intraoperative complications. However, it was not possible to match the plumb line at C7 and the center of the sacral vertical line (Figure 3c). Although slight PO remained postoperatively, the final standing posture and fatigability during walking improved dramatically.

Careful patient education was mandatory to prevent postoperative hip dislocation. Thoracolumbosacral orthosis immobilization was maintained for half a year after surgery. After gait rehabilitation for a few months, she had no difficulty in walking and also experienced indirect improvement of the severe knee pain at the final follow-up. Two years after surgery, spinal arthrodesis was completely accomplished by vigorous bone formation, without hip problems such as postoperative hip dislocation and loosening. Final radiographs showed improved alignment on sagittal and coronal planes (Figure 3c-3d).

Measurement methods of radiographic values associated with spinopelvic parameters were shown in Table 1. Perioperative radiographic values (preop/after THA/after ASD surgery/final follow-up) were shown in Table 2. In particular, preop. and final follow-up values were as follows: thoracolumbar Cobb angle (°): 40/25, lumbosacral Cobb angle (°): 60/14, C7 plumb line shift (mm): 24/0, PI: 61°, PT (°): 15/19, LL (°): 23/60, pelvic incidence minus lumbar lordosis (PI-LL): 38/1, the sagittal vertical axis (mm): 80/0, and PO (°): 28/15, respectively. The inclination angle of the acetabular cup changed from 52° to 35° after ASD surgery; however, the anteversion angle did not change (Figure 4a-4b). 

**Table 1:** Measurement of spinopelvic parameters.

Item	Measurement
C7 plumb line	A vertical line is drawn from the center of the C7 vertebral body on a posteroanterior standing whole spine radiograph.
Pelvic obliquity	The angle between lines 1 and 2 according to Osebold technique on a coronal plane. (Line 1 is drawn between the superior aspects of the iliac crests and line 2 is drawn parallel to the lower end of the radiograph.)
Lumbar lordosis	Cobb angle between a line along superior endplate of the L1 and a line along superior endplate of the S1, on a sagittal plane.
Sacral slope	The angle between a horizontal line and a line parallel to the superior endplate of S1 on a sagittal plane.
Pelvic tilt	The angle between a vertical line and a line bisecting S1 superior endplate and center of femoral heads on a sagittal plane.
Pelvic incidence	The angle between a line perpendicular to the midpoint of S1 superior endplate and a line bisecting the center of the femoral heads. Alternatively, the sum of pelvic tilt and sacral slope on a sagittal plane.
Anterior plane pelvic tilt	The angle between the two anterosuperior iliac spines and the anterior surface of the pubic symphysis.

**Table 2:** Radiographic parameters (preoperative, after THA, after scoliosis surgery, final follow-up). *Radiological Inclination angle was measured as an angle between lines 1 and 2. (Line 1 is drawn between the superior and inferior edges of the acetabular component, and line 2 is drawn across the bottom of the acetabular teardrops.) †Functional inclination angle was measured as an angle between lines 1 and 3. (Line 3 is drawn parallel to the lower end of the radiograph.)

	Preop.	After THA	After ASD surgery	Final follow-up
Thoracolumbar cobb angle (°)	40	40	19	25
Lumbosacral cobb angle (°)	60	60	22	14
C7 plumb line shift (mm)	24	13	0	0
Sagittal vertical axis (mm)	80	80	0	0
Pelvic tilt (°)	15	15	14	19
Pelvic incidence (°)	61	61	61	61
Lumbar lordosis (°)	23	23	66	60
PI-LL	38	38	-5	1
Pelvic obliquity (°)	28	19	15	15
Radiological inclination angle (°)*	34	30	30	30
Functional inclination angle (°)^†^	52	40	35	35
Anterior plane pelvic tilt (APPt) (°)	10	10	14	14

The Japanese Orthopaedic Association score of the left hip improved slightly from 61 to 69 points. The SRS-22 (function, pain, self-image, mental health, satisfaction) scores also changed 51 points from (2.8/1.4/1.8/4.2/none) preoperatively to 100 points (4.0/5.0/4.2/5.0/4.5) two years postoperatively. In particular, pain and self-image improved significantly, and the patient was satisfied with our surgical treatment.

## Discussion

In this report, following surgery, the infected hip was successfully fused and the infection was inactivated by appropriate chemotherapy; however, the flexion-adduction hip contracture unfortunately remained.

Hip spine syndrome

The term hip spine syndrome was introduced by Offierski and MacNab in 1983 [10]. They categorized this syndrome into four groups, as follows: (1) simple hip-spine syndrome with pathologic changes in the hip and spine with a clear source of pain and disability, (2) secondary hip-spine syndrome, defined as an aggravated spine syndrome consequent to hip deformity, (3) complex hip-spine syndrome, where both the spine and hip were symptomatic with no clear source of pain and disability, and (4) misdiagnosis syndrome, where the source of pain was not clearly identified. Our case may correspond to type (2).

There are some previous reports regarding the priority of treatment for patients with simultaneous hip and spinal disorders. With respect to the proper position of the acetabular cup, many authors have proposed spinal correction surgery for ASD prior to THA. Pelvic parameters have a significant influence on the position of the acetabular cup, which changes after spinal procedures [8]. Therefore, most surgeons recommend that the acetabular cup orientation should be adjusted after correcting spinal misalignment [7]. Zheng et al. directly compared the clinical impact of spinal osteotomy before THA with the impact of THA before spinal osteotomy in ankylosing spondylitis and recommended that spinal osteotomy should be performed prior to THA [11].

In contrast, Sultan et al. argued that hip surgery should be considered prior to spine surgery in cases with hip flexion contractures [9]. They reported that changes in spinal sagittal balance affect radiographic and clinical THA outcomes and that THA outcomes in lumbosacral fusion patients show an increased incidence of adverse events [9]. They also proposed that the presence of hip ﬂexion contractures must be addressed ﬁrst, and any spine deformity correction should not be performed before restoring normal hip mechanics by THA. They also recommended that spinal balance should be reevaluated after THA, and the necessity of spinal deformity corrective surgery should be considered accordingly.

Based on a functional study of preoperative standing and sitting lateral radiography views, Phan et al. proposed a guideline for surgeons who undertake THA during preoperative planning, to aid placement of the acetabular component in patients with spinal deformities. They proposed an algorithm based on flexible or rigid spinopelvic junctions, with appropriate sagittal spinal balance: PT<25°, PI-LL<10° or persistent imbalance, PT>25°, and PI-LL>10° [12]. For patients with rigid and unbalanced spines, more ideal cup positioning is necessary. They proposed placing the acetabular cup closer to the higher end of the safe zone. Owing to limited spinopelvic flexibility, such a cup would help compensate for the relative acetabular retroversion in the seated position while allowing a normal ROM in the standing position.

With regard to coronal balance, in our current case, if ASD surgery was performed before THA, her flexed-adducted contracted hip would be more severely adducted owing to correction of PO, which might have resulted in worsening of the coronal balance with hindering ambulation. Abe et al. reported the impact of leg-lengthening THA on the coronal alignment of the spine [13]. If the spine is flexible, the patient may show acceptable compensation in the lumbar or lumbosacral spine on a coronal plane after leg lengthening THA. In contrast, preoperative rigid scoliosis could have a risk in progress for spinal imbalance. On the other hand, in regard to sagittal balance, acquisition of sufficient lumbar lordosis after prior ASD surgery would align instrumented immobile trunk, resulting in difficulty of maintaining a sitting position until the next hip surgery. A similar case was previously reported by Yoshikawa et al. [14]. It would lead to sustained fatal complications such as deep venous thrombosis in the legs, aspiration pneumonia, decubitus ulcers, and mechanical loosening or dislodgement of spinal implants. Thus, we believe that according to an algorithm proposed by Sultan et al., hip surgery prioritized ASD surgery. In addition, as described above, we could reevaluate the entire standing coronal and sagittal spinal balance and remaining PO after THA surgery, without any perioperative complications.

Relationship between PO and spinal deformity

The relationship between PO and spinal deformity due to hip ankylosis is not fully understood. Morimoto et al. described the relationship between the coronal alignment of the lumbar spine and pelvis in patients with ankylosed hips [15]. They concluded that the abducted position is positively correlated with downward PO and the convexity of the lumbar scoliosis is toward the side of the ankylosed hip; in contrast, the adduction position is positively correlated with these results on the contralateral side. In our case, the ankylosed adducted right hip and ipsilateral upward PO led to reactive lumbosacral scoliosis with left-sided convexity, that gradually increased over a long period of time; this caused a compensatory thoracolumbar curve with right-sided convexity to maintain coronal balance. Since pelvic compensation for adjusting the LLD was inadequate in our current case, the patient had to compensate by keeping her contralateral knee extremely flexed. This compensation by the lower extremity was associated with fatigability of the quadriceps during gait; these defects should be treated surgically.

PO and anteversion of the acetabular component of the hip

PO is classified as suprapelvic, intrapelvic, and infrapelvic types; infrapelvic obliquity (IPO) occurs secondary to hip contractures. Both abduction and adduction hip contractures often lead to IPOs following secondary lumbosacral scoliosis. The angle of cup inclination in THA still remains within the “safe zone” of 5-25° anteversion with 30-50° inclination [16]. However, the effects of the actual mechanisms of IPOs on the position of the acetabular components remain unknown. Zhou et al. reported that the magnitude of the IPO had no significant effect on the anteversion of the cup, whereas a greater inclination angle of the cup was observed on postoperative radiographs in type 1C (abduction hip contracture and PO greater than 6°) cases; conversely, there were no significant changes in type 2C (adduction hip contracture and PO greater than 6°) cases [17]. They recommended a decrease in inclination by at least an 8° during cup insertion for IPOs of type 1C. Therefore, the inclination angle of the cup would increase as the pelvis became more horizontal in type 1C cases, whereas it would decrease in type 2C cases. In the current case, we speculated that in addition to cup inclination, anteversion after ASD surgery could decrease slightly due to reduction of PO and matching of PI and LL. Therefore, we placed an acetabular cup at 30° inclination, which is the lower limit of the safe zone in anticipation of incomplete PO reduction, and at 25° anteversion, which is the upper limit of the safe zone.

A retrospective review of a multicenter study of patients with THA prior to the spinal realignment procedure for ASD revealed that 1° of acetabular retroversion occurred with a 1.105° reduction in PT [8]. Cup anteversion and inclination angles in THA are directly influenced by PT. Whether PT changes before and after THA remains controversial; however, most studies have reported that THA-related changes in PT are considerably small [18,19]. Some studies have reported that specific groups of patients who are older and have disc space narrowing, lower lumbar lordosis, a wide contralateral joint space, or low back pain do not compensate using a rigid lumbar spine, but by using the pelvis, by changing PT [20,21].

## Conclusions

We report a case of secondary rigid lumbar scoliosis consequent to ankylosing TB hip in an elderly patient. THA and adductor tendonectomy were performed first, with positioning an acetabular cup at a small inclination angle and the upper limit of anteversion. Secondary, posterior spinal correction and fusion were performed from T10 to the pelvis without any perioperative complication. Lumbosacral Cobb angle, C7 plumb line shift, PI-LL value, and sagittal vertical axis improved dramatically after the surgery. Her symptoms including fatigability during walking resolved and the sagittal spinal balance on standing improved.

Based on a successful outcome in our case, we suggested that THA prior to spinal correction surgery may be a feasible option for treating rigid spinal deformities with hip ankylosis and pelvic obliquity.
